# Supplementary material for: Midbrain somatostatin-expressing cells control pain-suppression during defensive states
Source: eLife. 2026 Nov 10;14:RP106826. doi: 10.7554/eLife.106826 (PMC13750795; doi:10.7554/eLife.106826)
Supplement: Supplementary file 1 [file elife-106826-mdarchecklist1.docx]

Materials Design Analysis Reporting (MDAR) Checklist for Authors

**Winke et al., “Midbrain somatostatin-expressing cells control pain-suppression during defensive states”**

eLife — Version of Record. Corresponding author: Dr Cyril Herry, INSERM U1215, Neurocentre Magendie, University of Bordeaux.

*Section and item headings follow the MDAR checklist as published by eLife. Locations refer to the Version of Record manuscript unless stated otherwise. No entry is left outstanding.*

# Materials

| **Item** | **Reported / location** |
| --- | --- |
| Newly created materials | None. No new mouse line, plasmid, viral construct, antibody or cell line was generated. All viral vectors and probes are commercially available and identified below. |
| Antibodies | Not applicable to this study. No antibodies were used. |
| DNA and RNA sequences | No novel sequences. Commercial RNAscope probes, with catalogue numbers, are given in Methods, “Single-molecule fluorescent in situ hybridization”: Sst (ACDBio 404631), Slc32a1 (ACDBio 319191-C2), Slc17a6 (ACDBio 319171-C2), Cre (ACDBio 312281-C2), RNAscope Fluorescent Multiplex Kit (ACDBio 323110). |
| Cell materials | Not applicable to this study. No cell lines or primary cultures were used. |
| Experimental animals | Species: Mus musculus. Sex: male only, for every experiment. Age: 8–14 weeks; stereotaxic injections at 8–9 weeks.  Strains and genetic status: male C57BL/6J (Janvier Labs); Sst-IRES-Cre, heterozygous and homozygous (The Jackson Laboratory, stock #013044, mixed background); Vip-IRES-Cre, heterozygous (The Jackson Laboratory, stock #010908).  Housing: individually housed, 12 h light–dark cycle, food and water ad libitum.  Reported in Methods, “Animals”. Note: the Reviewed Preprint cited stock #012712 for the Sst-IRES-Cre line; the correct stock is #013044 and the line is on a mixed background. This is corrected in the Version of Record and is listed in the accompanying declaration of modifications.  The restriction to male mice is stated in the Abstract and its consequence for generality is discussed explicitly in the Discussion. |
| Viral vectors | AAV5-EF1a-DIO-hChR2(H134R)-EYFP, titre 3.2×10¹² (Vector Core, University of North Carolina); AAV9-CAG-FLEX-ArchT-GFP, titre 4.7×10¹² (UNC); AAV5-FLEX-GFP, titre 4.5×10¹² (UNC). Volumes, coordinates and timings in Methods, “Surgery and optogenetics”. |
| Plants and microbes | Not applicable to this study. |
| Human research participants | Not applicable to this study. |

# Design

| **Item** | **Reported / location** |
| --- | --- |
| Study protocol / pre-registration | Not pre-registered. Not a clinical trial, so no trial registration number. |
| Laboratory protocol | No separate protocol deposit (protocols.io or equivalent). All procedures are described step by step in Methods. The cued fear-conditioned analgesia (CFCA) paradigm is newly developed for this study and is described in full, including apparatus, contexts, stimulus parameters and timings. |
| Sample size determination | No statistical method was used to predetermine sample sizes; sizes were based on previous studies in the laboratory. Stated verbatim in Methods, “Statistics”. No formal power calculation was performed. |
| Randomisation | Animals were not allocated to opsin groups by a formal randomisation procedure. Within-subject order was counterbalanced within each group: the CS− and CS+ hot-plate trials were counterbalanced (Methods, “CFCA”), as was the stimulated compartment in the real-time place preference assay (Methods, “Real-time Place Preference assay”). |
| Blinding | Blinding to the opsin was applied for the optogenetic experiments (Methods, “Statistics”). The in vivo electrophysiological recordings were not blinded. |
| Inclusion and exclusion criteria | Pre-established, from preliminary data: mice proceeded to the FC hot-plate test only if the discrimination index was ≥ 0.4 and the conditioning index ≥ 0.3. Mice failing these criteria after a second conditioning session were discarded. Mice with an incorrect opsin injection or a misplaced optic fibre were discarded. The Methods state explicitly that no other mice were excluded.  For single-unit recordings, outliers were removed using the Tukey method (Methods, “Statistics”).  In the deposited source data, values excluded from an analysis are retained as rows and flagged in an included_in_analysis column with the reason, rather than deleted (Figure 4c and Figure 5f right). |
| Sample definition and replication | The experimental unit is the mouse for all behavioural data and for the spinal field-potential recordings, and the cell for the wide dynamic range (WDR) single-unit recordings, where the number of contributing mice is also given.  For single-unit recordings, each cell's value is the average of four electrical stimuli — a technical average within one cell, not an independent replicate (Methods, “Statistics”).  n is given in every figure legend, in Supplementary File 1 and in the header of every deposited source-data file. The experimental unit is named explicitly in each of those headers. |

# Ethics

| **Item** | **Reported / location** |
| --- | --- |
| Human participants | Not applicable to this study. |
| Experimental animals | All procedures were performed in accordance with standard ethical guidelines (European Communities Directive 86/60-EEC) and were approved by the Committee on Animal Health and Care of the Institut National de la Santé et de la Recherche Médicale (INSERM) and the French Ministry of Agriculture and Forestry, agreement #A3312001. Reported in Methods, “Animals”. Analgesia was administered during surgery (Metacam, Boehringer; Lurocaine, Vetoquinol) and mice recovered for at least five days. |
| Specimens and field samples | Not applicable to this study. |
| Dual Use Research of Concern | Not applicable to this study. |

# Analysis

| **Item** | **Reported / location** |
| --- | --- |
| Attrition | Exclusion criteria were pre-established and are stated in Methods (see Design, above), together with the explicit statement that no other mice were excluded. The numbers of animals included are reported per experiment in the figure legends, and the optic fibre placement maps report the totals verified histologically: 39 Sst-IRES-Cre mice (Figure 2—figure supplement 1) and 10 Vip-IRES-Cre mice (Figure 2—figure supplement 6). Individual data points omitted from an analysis are flagged in the deposited data rather than removed. |
| Statistics | Every test, test statistic, exact P value, post-hoc comparison and n is reported in Supplementary File 1, and the test is named in each figure legend.  Normality was tested with the Kolmogorov–Smirnov test and non-parametric tests were used for non-normally distributed data (Methods, “Statistics”).  Analyses were performed in GraphPad Prism; part of the earlier behavioural analyses were performed in StatView, and the corresponding analysis files are the source of the deposited values.  Box-whisker plots show the median, interquartile range and 5th–95th percentiles. Significance is reported as *P<0.05, **P<0.01, ***P<0.001, ****P<0.0001.  Every deposited value table was independently checked twice: the panel was reproduced from it and the published test statistic was recomputed from it. The recomputed statistic is recorded in each file header, and any difference from the published value is stated there with its cause. |

# Data availability

| **Item** | **Reported / location** |
| --- | --- |
| Data deposition | All source data underlying the quantitative panels are deposited in the Dryad Digital Repository, DOI https://doi.org/10.5061/dryad.63xsj3vj3. The deposit contains 56 CSV files, one per panel, named with the eLife panel designation, plus a README describing every file, its columns, units, experimental unit, sample size and the statistic recomputed from it. |
| Restrictions on access | None. The data are released without restriction. |
| Data not deposited | One panel is absent: Figure 5—figure supplement 1b (GFP control for the vlPAG-to-RVM pathway, three fibre components, 7 cells, 3 mice). Its value table could not be located. This is stated in the deposit README, together with the published statistics for that panel. |
| Reused datasets | None. No previously published dataset was reanalysed. |

# Code availability

| **Item** | **Reported / location** |
| --- | --- |
| Newly generated code | TestoFreez, the custom code for automated detection of freezing from thermographic recordings, is available at https://github.com/djercog/TestoFreez, released under the MIT License (LICENSE.txt in the repository, Copyright (c) 2026 Daniel Jercog). Cited in Methods and in the reference list. No specific commit or tagged release is cited: the reference points to the repository itself. The in-house MATLAB routines used to derive distance travelled from idTracker output are not deposited; the values they produced are in the deposited source data (Figure 2-figure supplement 3c and 3d). |
| Third-party software | idTracker (cited in Methods) for video tracking; MATLAB (The MathWorks) for analysis; ImageJ for smFISH quantification; GraphPad Prism and StatView for statistics; Imetronic software for behavioural apparatus control and for permanence time in the place preference assay. |

# Outstanding items

One entry above cannot be completed until the corresponding step is done.

| **Item** | **Reported / location** |
| --- | --- |
| Dryad DOI | Obtained on 6 October 2026: https://doi.org/10.5061/dryad.63xsj3vj3. The dataset has been submitted to Dryad and is in curation; the DOI is reserved and becomes active when the dataset is published. It is inserted in the Data availability statement of the manuscript. |

*Resolved on 5 October 2026: the TestoFreez licence (MIT, LICENSE.txt added to the repository by the code author) and the question of a pinned version, which was dropped — the reference cites the repository rather than a commit or tag.*
