# Supplementary material for: Midbrain somatostatin-expressing cells control pain-suppression during defensive states
Source: eLife. 2026 Nov 10;14:RP106826. doi: 10.7554/eLife.106826 (PMC13750795; doi:10.7554/eLife.106826)
Supplement: Supplementary file 2 [file elife-106826-supp1.docx]

| **Figure**  **number** | **Normality test** | **Statistic method** | **Main effect** | **Post hoc multiple comparisons test** | **Number of**  **mice** | **Number of**  **cells** |
| --- | --- | --- | --- | --- | --- | --- |
| 1b | Passed | One-way RM ANOVA followed by Bonferroni post-hoc test | F_(2, 22)_ = 160.8  P value < 0.0001 | BL vs. CS^-^, P > 0.9999, ns  BL vs. CS^+^, P < 0.0001  CS^-^ vs. CS^+^, P < 0.0001 | 12 | NA |
| 1e | Failed | Wilcoxon matched-pairs signed rank test (two-tailed) | P = 0.0005 | *W* = 78.00 | 12 | NA |
| 1f | Onset, failed  Offset, failed | Wilcoxon matched-pairs signed rank test (two-tailed) | Onset, P = 0.0015  Offset, P = 0.8330, ns | Onset, *W* = 74  Offset, *W* = 6 | 12 | NA |
| 1g | Failed | Friedman test followed by Dunn's multiple post-hoc test | P value < 0.0001  *Q* = 48.13 | BL vs. CS^-^, P > 0.9999, ns  BL/CS^-^ vs. CS^+^_1st block_, P < 0.0001  CS^-^ vs. CS^+^_5th/6th block_, P > 0.9999, ns  CS^+^_1st block_ vs. CS^+^_5th/6th block_, P < 0.005 | 10 | NA |
| 1h | Passed | Paired t-test (two-tailed) | P = 0.4365, ns | *t*_9_ = 0.8143 | 10 | NA |
| 1i | Failed | Kruskal-Wallis test followed by Dunn's multiple post-hoc test | P < 0.0001  *H* = 25.32 | No tone vs. Tone, P = 0.7710, ns  No tone vs. CS^-^, P > 0.9999, ns  Tone vs. CS^-^, P > 0.9999, ns  No tone vs. CS^+^, P < 0.0001  Tone vs. CS^+^, P = 0.0009  CS^-^ vs CS^+^, P = 0.0033 | No tone = 10  Tone = 12  CSs = 12 | NA |
| 2c | Passed | One-way RM ANOVA followed by Bonferroni post-hoc test | F_(2,30)_ = 396.8  P < 0.0001 | BL vs. CS^-^, P > 0.9999, ns  BL vs. CS^+^, P < 0.0001  CS^-^ vs. CS^+^, P < 0.0001 | 16 | NA |
| 2d | Passed | Two-way RM ANOVA followed by Bonferroni post-hoc test | Opsin x CS, F_(1,14)_ = 8.514  P = 0.0112 | CS^-^ vs. CS^+^, GFP, P = 0.0003  CS^-^ vs. CS^+^, ChR2, P = 0.3876, ns  GFP vs. ChR2, CS^-^, P > 0.9999, ns  GFP vs. ChR2, CS^+^, P = 0.0344 | GFP = 7  ChR2 = 9 | NA |
| 2e | Passed | One-way RM ANOVA followed by Bonferroni post-hoc test | F_(2,44)_ = 280.4  P < 0.0001 | BL vs. CS^-^, P = 0.2337, ns  BL vs. CS^+^, P < 0.0001  CS^-^ vs. CS^+^, P < 0.0001 | 23 | NA |
| 2f | Passed | Two-way RM ANOVA followed by Bonferroni post-hoc test | Opsin x CS, F_(1,21)_ = 10.64  P = 0.0037 | CS^-^ vs. CS^+^, GFP, P = 0.0003  CS^-^ vs. CS^+^, ArchT, P > 0.9999, ns  GFP vs. ArchT, CS^-^, P < 0.0001  GFP vs. ArchT, CS^+^, P = 0.0265 | GFP = 12  ArchT = 11 | NA |
| 3c | Passed | Two-way RM ANOVA followed by Bonferroni post-hoc test | Opsin x CS, F_(4,48)_ = 0.1954  P = 0.9396, ns | BL vs. CS^-^, GFP/ArchT, P > 0.9999, ns  BL vs. CS^+^_1st/2nd/3rd_, GFP/ArchT, P < 0.0001  CS^-^ vs. CS^+^_1st/2nd/3rd_, GFP/ArchT, P < 0.0001  CS^+^_1st_ vs. CS^+^_2nd/3rd_, GFP/ArchT, P > 0.9999, ns  CS^+^_2nd_ vs. CS^+^_3rd_, GFP/ArchT, P > 0.9999, ns  GFP vs. ArchT, BL/CS^-^/CS^+^_1st/2nd/3rd_, P > 0.9999, ns | GFP = 6  ArchT = 8 | NA |
| 3d | Passed | Two-way RM ANOVA followed by Bonferroni post-hoc test | Opsin x CS, F_(4,60)_ = 7.957  P < 0.0001 | BL vs. CS^-^, GFP/ChR2, P > 0.9999, ns  BL vs. CS^+^_1st/2nd/3rd_, GFP, P < 0.0001  CS^-^ vs. CS^+^_1st/2nd/3rd_, GFP, P < 0.0001  CS^+^_1st_ vs. CS^+^_2nd/3rd_, GFP, P > 0.9999, ns  CS^+^_2nd_ vs. CS^+^_3rd_, GFP, P > 0.9999, ns  BL vs. CS^+^_1st/3rd_, ChR2, P < 0.0001  BL vs. CS^+^_2nd_, ChR2, P = 0.0789, ns  CS^-^ vs. CS^+^_1st/3rd_, ChR2, P < 0.0001  CS^-^ vs. CS^+^_2nd_, ChR2, P > 0.9999, ns  CS^+^_1st_ vs. CS^+^_2nd_, ChR2, P < 0.0001  CS^+^_1st_ vs. CS^+^_3rd_, ChR2, P = 0.2372, ns  CS^+^_2nd_ vs. CS^+^_3rd_, ChR2, P < 0.0001  GFP vs. ChR2, BL/CS^-^/CS^+^_1st/3rd_, P > 0.9999, ns  GFP vs. ChR2, CS^+^_2nd_, P = 0.0002 | GFP = 9  ChR2 = 8 | NA |
| 4b | Passed | One-way RM ANOVA followed by Bonferroni post-hoc test | F_(2, 14)_ = 17.94  P = 0.0001 | OFF1 vs. ON, P = 0.0006  OFF2 vs. ON, P = 0.0003  OFF1 vs. OFF2, P > 0.9999, ns | 8 | NA |
| 4c | Passed | One-way RM ANOVA followed by Bonferroni post-hoc test | F_(2, 8)_ = 5.826  P = 0.0275 | OFF1 vs. ON, P = 0.0414  OFF2 vs. ON, P = 0.0452  OFF1 vs. OFF2, P = 0.9980, ns | 5 | NA |
| 4d | A-β fiber, passed  A-δ fiber, passed  C fiber, passed | Paired t-test (two-tailed) | A-β fiber, P = 0.5430, ns  A-δ fiber, P = 0.0022  C fiber, P = 0.0033 | A-β fiber, *t*_11_ = 0.6319  A-δ fiber, *t*_11_ = 3.959  C fiber, *t*_11_ = 3.739 | 8 | A-β fiber, 12  A-δ fiber, 12  C fiber, 12 |
| 4e | A-β fiber, passed  A-δ fiber, passed  C fiber, passed | Paired t-test (two-tailed) | A-β fiber, P = 0.1386, ns  A-δ fiber, P = 0.0241  C fiber, P < 0.0001 | A-β fiber, *t*_13_ = 1.578  A-δ fiber, *t*_13_ = 2.553  C fiber, *t*_13_ = 6.274 | 5 | A-β fiber, 14  A-δ fiber, 14  C fiber, 14 |
| 4f | A-β fiber, failed  A-δ fiber, failed  C fiber, failed | Wilcoxon matched-pairs signed rank test (two-tailed) | A-β fiber, P = 0.3750, ns  A-δ fiber, P = 0.0156  C fiber, P = 0.0020 | A-β fiber, *W* = 7.0  A-δ fiber, *W* = 28.0  C fiber, *W* = 55.0 | 8 | A-β fiber, 10  A-δ fiber, 10  C fiber, 10 |
| 5e | Passed | Paired t-test (two-tailed) | P = 0.3683, ns | *t*_10_ = 0.94222 | 7 | 11 |
| 5f, left | A-β fiber, passed  A-δ fiber, passed  C fiber, failed | Paired t-test (two-tailed)  Paired t-test (two-tailed)  Wilcoxon matched-pairs signed rank test (two-tailed) | A-β fiber, P = 0.0740, ns  A-δ fiber, P = 0.0346  C fiber, P = 0.0020 | A-β fiber, *t*_9_ = 2.021  A-δ fiber, *t*_9_ = 2.487  C fiber, *W* = 55.0 | 7 | A-β fiber, 10  A-δ fiber, 10  C fiber, 10 |
| 5f, right | Failed | Wilcoxon matched-pairs signed rank test (two-tailed) | P = 0.0078 | *W* = 36.0 | 7 | 8 |
| 6b | Passed | Two-way RM ANOVA followed by Bonferroni post-hoc test | Opsin x CS, F_(4,44)_ = 0.6292  P = 0.6443, ns | BL vs. CS^-^, GFP/ChR2, P > 0.9999, ns  BL vs. CS^+^_1st/2nd/3rd_, GFP/ChR2, P < 0.0001  CS^-^ vs. CS^+^_1st/2nd/3rd_, GFP/ChR2, P < 0.0001  CS^+^_1st_ vs. CS^+^_2nd_, GFP/ChR2, P > 0.9999, ns  CS^+^_2nd_ vs. CS^+^_3rd_, GFP/ChR2, P > 0.9999, ns  CS^+^_1st_ vs. CS^+^_3rd_, GFP, P = 0.0090  CS^+^_1st_ vs. CS^+^_3rd_, ChR2, P = 0.0003  GFP vs. ChR2, BL/CS^-^/CS^+^_1st/2nd/3rd_, P > 0.9999, ns | GFP = 7  ChR2 = 6 | NA |
| 6c | Passed | One-way RM ANOVA followed by Bonferroni post-hoc test | F_(2,26)_ = 274.2  P < 0.0001 | BL vs. CS^-^, P > 0.9999, ns  BL vs. CS^+^, P < 0.0001  CS^-^ vs. CS^+^, P < 0.0001 | 14 | NA |
| 6d | Passed | Two-way RM ANOVA followed by Bonferroni post-hoc test | Opsin x CS, F(1,12) = 19.87  P = 0.0008 | CS^-^ vs. CS^+^ - GFP, P < 0.0001  CS^-^ vs. CS^+^ - ChR2, P > 0.9999, ns  GFP vs. ChR2 - CS-, P = 0.1794, ns  GFP vs. ChR2 - CS+, P < 0.0001 | GFP = 7  ChR2 = 7 | NA |
| 1—fs1a | Failed | Friedman test followed by Dunn's multiple post-hoc test | P = 0.0003  *Q* = 16.55 | BL vs. CS^-^, P = 0.3074, ns  BL vs. CS^+^, P = 0.0003  CS^-^ vs. CS^+^, P = 0.0742, ns | 12 | NA |
| 1—fs1b | Failed | Wilcoxon matched-pairs signed rank test (two-tailed) | P = 0.0005 | *W* = 78.0 | 12 | NA |
| 1—fs1d | Passed | Mixed-effects model followed by Bonferroni post-hoc test | CS x Time, F_(9,238)_ = 0.1716  P = 0.9967 | CS^+^ vs. CS^-^ for each time point, all P > 0.9999, ns | 13 | NA |
| 1—fs1e | Passed | Mixed-effects model followed by Bonferroni post-hoc test | CS x Time, F_(9,238)_ = 0.7147  P = 0.6952 | CS^+^ vs. CS^-^ for each time point, all P > 0.9999, ns | 13 | NA |
| 1—fs2b | No tone, passed  Tone, passed  CFCA, failed | Paired t-test (two-tailed)  Paired t-test (two-tailed)  Wilcoxon matched-pairs signed rank test (two-tailed) | No tone, P = 0.9866, ns  Tone, P = 0.9085, ns  CFCA, P = 0.0005 | No tone, *t*_9_ = 0.01732  Tone, *t*_11_ = 0.1176  CFCA, *W* = 78.0 | No tone = 10  Tone = 12  CFCA = 12 | NA |
| 1—fs2b | Failed | Kruskal-Wallis test followed by Dunn's multiple post-hoc test | P = 0.0001  *H* = 25.45 | No tone 1 vs. No tone 2/Tone 1/Tone 2/CS^-^, P > 0.9999, ns  No tone 2 vs. Tone 1/Tone 2/CS^-^, P > 0.9999, ns  Tone 1 vs. Tone 2/CS^-^, P > 0.9999, ns  Tone 2 vs. Tone CS^-^, P > 0.9999, ns  No tone 1 vs. CS^+^, P = 0.0002  No tone 2 vs. CS^+^, P = 0.0009  Tone 1 vs. CS^+^, P = 0.0178  Tone 2 vs. CS^+^, P = 0.0133  CS^-^ vs. CS^+^, P = 0.0082 | No tone = 10  Tone = 12  CFCA = 12 | NA |
| 1—fs2c | No tone, passed  Tone, failed  CFCA, failed | Paired t-test (two-tailed)  Wilcoxon matched-pairs signed rank test (two-tailed) | No tone, P = 0.6216, ns  Tone, P = 0.4375, ns  CS, P = 0.0005 | No tone, *t*_9_ = 0.5111  Tone, *W* = -21.0  CFCA, *W* = 78.0 | No tone = 10  Tone = 12  CFCA = 12 | NA |
| 1—fs2c | Failed | Kruskal-Wallis test followed by Dunn's multiple post-hoc test | P < 0.0001  *H* = 29.30 | No tone 1 vs. No tone 2/Tone 1/Tone 2/CS^-^, P > 0.9999, ns  No tone 2 vs. Tone 1/Tone 2/CS^-^, P > 0.9999, ns  Tone 1 vs. Tone 2/CS^-^, P > 0.9999, ns  Tone 2 vs. Tone CS^-^, P > 0.9999, ns  No tone 1 vs. CS^+^, P < 0.0001  No tone 2 vs. CS^+^, P = 0.0010  Tone 1 vs. CS^+^, P = 0.0339  Tone 2 vs. CS^+^, P = 0.0037  CS^-^ vs. CS^+^, P = 0.0033 | No tone = 10  Tone = 12  CFCA = 12 | NA |
| 1—fs2d | Passed | Paired t-test (two-tailed) | P = 0.3276, ns | *t*_9_ = 1.035 | 10 | NA |
| 1—fs2f | Passed | One-way RM ANOVA followed by Bonferroni post-hoc test | F_(2,18)_ = 558.2  P < 0.0001 | BL vs. CS^-^, P > 0.9999, ns  BL vs. CS^+^, P < 0.0001  CS^-^ vs. CS^+^, P < 0.0001 | 10 | NA |
| 1—fs2g | Passed | One-way RM ANOVA followed by Bonferroni post-hoc test | F_(2,18)_ = 56.00  P < 0.0001 | BL vs. CS^-^, P > 0.9999, ns  BL vs. CS^+^, P < 0.0001  CS^-^ vs. CS^+^, P < 0.0001 | 10 | NA |
| 1—fs2h, left | Passed | Paired t-test (two-tailed) | P = 0.0029 | *t*_9_ = 4.054 | 10 | NA |
| 1—fs2h, right | Passed | Paired t-test (two-tailed) | P = 0.0006 | *t*_9_ = 5.184 | 10 | NA |
| 1—fs2i, left | Passed | Paired t-test (two-tailed) | P = 0.0362 | *t*_9_ = 2.459 | 10 | NA |
| 1—fs2i, right | Passed | Paired t-test (two-tailed) | P = 0.0245 | *t*_9_ = 2.697 | 10 | NA |
| 1—fs3c | NA | Cell counts, no statistical test applied | dlPAG, ~94% (101/107) and ~84% (101/120)  vlPAG, ~88% (74/84) and ~99% (74/75) | NA | NA | dlPAG = 107 / 120  vlPAG = 84 / 75 |
| 1—fs3e | NA | Cell counts, no statistical test applied | dlPAG, ~21% (17/80) and ~19% (17/88)  vlPAG, ~98% (183/186) and ~72% (183/254) | NA | NA | dlPAG = 80 / 88  vlPAG = 186 / 254 |
| 1—fs3g | NA | Cell counts, no statistical test applied | dlPAG, ~89% (54/61) and ~31% (54/172)  vlPAG, ~24% (43/176) and ~37% (43/115) | NA | NA | dlPAG = 61 / 172  vlPAG = 176 / 115 |
| 2—fs1 | NA | No statistical test (anatomical verification of optic fiber placement) | NA | NA | 39 | NA |
| 2—fs2a | Passed | Unpaired t-test (two-tailed) | P = 0.6052 | *t*_14_ = 0.5288 | GFP = 7  ChR2 = 9 | NA |
| 2—fs2b | Passed | Unpaired t-test (two-tailed) | P = 0.4575 | *t*_21_ = 0.7569 | GFP = 12  ArchT = 11 | NA |
| 2—fs2c | Passed | Unpaired t-test (two-tailed) | P = 0.2333 | *t*_14_ = 1.246 | GFP = 7  ChR2 = 9 | NA |
| 2—fs2d | Passed | Unpaired t-test (two-tailed) | P = 0.1320 | *t*_21_ = 1.567 | GFP = 12  ArchT = 11 | NA |
| 2—fs3a | Passed | Two-way RM ANOVA followed by Bonferroni post-hoc test | Opsin x CS, F_(1,14)_ = 11.40  P = 0.0045 | CS^-^ vs. CS^+^, GFP, P < 0.0001  CS^-^ vs. CS^+^, ChR2, P = 0.1783, ns  GFP vs. ChR2, CS^-^, P > 0.9999, ns  GFP vs. ChR2, CS^+^, P = 0.0322 | GFP = 7  ChR2 = 9 | NA |
| 2—fs3b | Passed | Two-way RM ANOVA followed by Bonferroni post-hoc test | Opsin x CS, F_(1,21)_ = 10.84  P = 0.0035 | CS^-^ vs. CS^+^, GFP, P = 0.0003  CS^-^ vs. CS^+^, ArchT, P > 0.9999, ns  GFP vs. ArchT, CS^-^, P < 0.0001  GFP vs. ArchT, CS^+^, P = 0.0704, ns | GFP = 12  ArchT = 11 | NA |
| 2—fs3c | Passed | Two-way RM ANOVA followed by Bonferroni post-hoc test | Opsin x Light, F_(2,28)_ = 0.6855  P = 0.5121, ns | OFF1 vs. ON, GFP/ChR2, P > 0.9999, ns  OFF2 vs. ON, GFP/ChR2, P > 0.9999, ns  OFF1 vs. OFF2, GFP/ChR2, P > 0.9999, ns  GFP vs. ChR2, OFF1/ON/OFF2, P > 0.9999, ns | GFP = 7  ChR2 = 9 | NA |
| 2—fs3d | Passed | Two-way RM ANOVA followed by Bonferroni post-hoc test | Opsin x Light, F_(2,42)_ = 0.9243  P = 0.4047, ns | OFF1 vs. ON, GFP, P = 0.0165  OFF1 vs. ON, ArchT, P = 0.0003  OFF2 vs. ON, GFP/ArchT, P > 0.9999, ns  OFF1 vs. OFF2, GFP/ArchT, P > 0.9999, ns  GFP vs. ArchT, OFF1/ON/OFF2, P > 0.9999, ns | GFP = 12  ArchT = 11 | NA |
| 2—fs3f | Failed | Mann-Whitney test (two-tailed) | P = 0.1812 | *U* = 13 | GFP = 6  ChR2 = 8 | NA |
| 2—fs4b | Passed | Two-way RM ANOVA followed by Bonferroni post-hoc test | Opsin x CS, F_(2,22)_ = 0.7582  P = 0.4804, ns | GFP vs. ChR2, BL/CS^-^/CS^+^, P > 0.9999, ns | GFP = 6  ChR2 = 7 | NA |
| 2—fs4c | Passed | Two-way RM ANOVA followed by Bonferroni post-hoc test | Opsin x CS, F_(2,22)_ = 1.926  P = 0.1695, ns | GFP vs. ChR2, BL/CS^-^/CS^+^, P > 0.9999, ns | GFP = 6  ChR2 = 7 | NA |
| 2—fs4d | Passed | Two-way RM ANOVA followed by Bonferroni post-hoc test | Opsin x CS, F_(2,22)_ = 0.1444  P = 0.8663, ns | GFP vs. ChR2, BL/CS^-^/CS^+^, P > 0.9999, ns | GFP = 6  ChR2 = 7 | NA |
| 2—fs4e | Passed | Two-way RM ANOVA followed by Bonferroni post-hoc test | Opsin x CS, F_(2,22)_ = 0.1095  P = 0.8967, ns | GFP vs. ChR2, BL/CS^-^/CS^+^, P > 0.9999, ns  BL vs. CS^-^, GFP, P > 0.9999, ns  BL vs. CS^-^, ChR2, P > 0.5328, ns  BL vs. CS^+^, GFP/ChR2, P < 0.0001  CS^-^ vs. CS^+^, GFP/ChR2, P < 0.0001 | GFP = 6  ChR2 = 7 | NA |
| 2—fs5c | Failed | Friedman test followed by Dunn's multiple post-hoc test | P value < 0.0001  *Q* = 19.85 | BL vs. CS^-^, P > 0.9999, ns  BL vs. CS^+^, P = 0.0012  CS^-^ vs. CS^+^, P = 0.0001 | 13 | NA |
| 2—fs5d | Passed | Two-way RM ANOVA followed by Bonferroni post-hoc test | Opsin x CS, F_(1,11)_ = 12.39  P = 0.0048 | CS^-^ vs. CS^+^, GFP, P = 0.0001  CS^-^ vs. CS^+^, ChR2, P = 0.3342, ns  GFP vs. ChR2, CS^-^, P > 0.9999, ns  GFP vs. ChR2, CS^+^, P = 0.0300 | GFP = 6  ChR2 = 7 | NA |
| 2—fs5g | Passed | One-way RM ANOVA followed by Bonferroni post-hoc test | F_(2,18)_ = 147.1  P < 0.0001 | BL vs. CS^-^, P = 0.8655, ns  BL vs. CS^+^, P < 0.0001  CS^-^ vs. CS^+^, P < 0.0001 | 10 | NA |
| 2—fs5h | Passed | Two-way RM ANOVA followed by Bonferroni post-hoc test | Opsin x CS, F_(1,8)_ = 0.3984  P = 0.5455, ns | CS^-^ vs. CS^+^, GFP, P = 0.0001  CS^-^ vs. CS^+^, ArchT, P = 0.0003  GFP vs. ArchT, CS^-^, P > 0.9999, ns  GFP vs. ArchT, CS^+^, P = 0.3869, ns | GFP = 5  ArchT = 5 | NA |
| 2—fs6 | NA | No statistical test (anatomical verification of optic fiber placement) | NA | NA | 10 | NA |
| 4—fs1c | Passed | Paired t-test (two-tailed) | P = 0.6929, ns | *t*_4_ = 0.4247 | 5 | 5 |
| 5—fs1b | A-β fiber, passed  A-δ fiber, passed  C fiber, passed | Paired t-test (two-tailed) | A-β fiber, P = 0.3750, ns  A-δ fiber, P = 0.7369, ns  C fiber, P = 0.4150, ns | A-β fiber, *t*_6_ = 0.9564  A-δ fiber, *t*_6_ = 0.3520  C fiber, *t*_6_ = 0.8755 | 3 | A-β fiber, 7  A-δ fiber, 7  C fiber, 7 |
