# Supplementary material for: Midbrain somatostatin-expressing cells control pain-suppression during defensive states
Source: eLife. 2026 Nov 10;14:RP106826. doi: 10.7554/eLife.106826 (PMC13750795; doi:10.7554/eLife.106826)
Supplement: Peer review history

# Editor report: eLife Assessment

**DOI:** 10.7554/eLife.106826.3.sa0

**Authors:** Michael A McDannald

**Affiliations:** Boston College United States

**Keywords:** Exceptional, Important

This **important** study shows that long-range somatostatin-expressing neurons in the ventrolateral periaqueductal grey that project to the rostral ventromedial medulla selectively suppress pain responses during conditioned fear. The evidence supporting these conclusions is **exceptional**, with methods spanning a novel cued fear-conditioned analgesia paradigm, cell-type-specific optogenetic activation and inhibition, anatomical circuit tracing, and in vivo spinal cord electrophysiology. These results will be of broad interest to systems and behavioral neuroscientists studying fear, pain, and descending pain-control circuitry.

# Referee report: Reviewer #1 (Public review)

**DOI:** 10.7554/eLife.106826.3.sa1

[Editors' note: this version has been assessed by the Reviewing Editor without further input from the original reviewers. The authors have addressed the comments raised in the previous round of review.]

Summary:

In the manuscript by Winke et al, the authors present evidence that fear-induced analgesia is mediated by somatostatin projection cells from the vlPAG to the RVM. This study uses a mouse model of fear-induced analgesia, and incorporates optogenetic circuit manipulation with behaviour and electrophysiology to gain a meaningful insight into a novel circuit involved in fear-induced analgesia.

Strengths:

(1) This is a well-constructed study with appropriate controls and analyses.

(2) Alternative interpretations of the data are systematically considered and eliminated via rational experiments. The authors are commended for a nice piece of experimental work.

(3) The vlPAG is a known region of pain modulation, and this study adds valuable insight to the circuit involved in fear-associated analgesia.

Weaknesses:

Only male mice are included in this study. [This has been explained and noted as a limitation.]

# Referee report: Reviewer #2 (Public review)

**DOI:** 10.7554/eLife.106826.3.sa2

Summary:

Wenke et al. investigated the role of vlPAG somatostatin-expressing neurons in the mediation of analgesia during defensive states. A newly developed paradigm of cued fear-conditioned analgesia, which consists of a combination of an auditory fear retrieval session and a pain test, was used to evaluate this cell population's contribution to fear-mediated analgesia. Optogenetic manipulation of vlPAG SST+ neurons modulated the responses to a nociceptive cue (Hot Plate) presented concomitantly with an aversively conditioned tone. At the same time, alterations in the freezing levels could be observed during optogenetic activation of vlPAG SST+ neurons. In order to disentangle the impact of these cells on analgesia from their impact on the expression of defensive behaviors, the authors performed electrophysiological recordings from the dorsal horn in the spinal cord of anesthetized mice. A vlPAG-RVM-DH pathway was identified to trigger nociceptive C-fibers upon optic activation of the RVM. Finally, pathway-specific activation of SST+ vlPAG-RVM neurons could abolish CS-induced analgesia.

Strengths:

The study addresses a relevant topic, that is, brainstem circuits for pain-modulatory mechanisms as part of defensive states evoked by threat. This is important because the circuit mechanisms underlying pain are still not fully understood, and defining molecular markers of cellular circuit substrates may support the identification of potential pharmaceutical targets in treating pain. The authors confirm a previous study in that a somatostatin-positive cellular population presents a crucial vlPAG circuit element mediating anti-nociceptive effects. Key novelty aspects of the present study are the demonstration that these neurons seem to play a role specifically in threat-induced analgesia. This was possible by the elegant design and application of a novel fear analgesia paradigm, combined with cell- and pathway-specific optogenetics.

# Referee report: Reviewer #3 (Public review)

**DOI:** 10.7554/eLife.106826.3.sa3

Summary:

Conditioned analgesia refers to the ability of a learned fear cue to suppress pain-related behavior and neural activity. Understudied, the authors developed a novel conditioned analgesia procedure in which a cue that had been paired or unpaired with shock was played while a hot plate increased temperature. Compared to several control conditions, the authors found increased latency to a nociceptive response (paw licking). The authors identified somatostatin neurons in the periaqueductal gray as a likely mediator of the behavior. They then showed that: (1) stimulating vlPAG-SST neurons blocked nociceptive response latency increases to the CS+, (2) stimulating vlPAG-SST neurons suppressed fear retrieval freezing, (3) stimulating vs. inhibiting vlPAG-SST neurons drove opposing modulation of c-fibers and Aδ-fibers, (4) direct-projecting vlPAG SST neurons modulate freezing while RVM-projecting vlPAG SST neurons modulate conditioned analgesia.

Strengths:

These experiments have many strengths. The behavioral assay is chief among them. The assay is robust and controls for confounding factors to reveal a repeatable effect of a shock-paired cue to delay nociceptive responding. The optogenetic experiments provide the correct level of temporal precision, given the authors' time-specific interest in cued responding. Combining neuronal manipulations with spinal recordings is particularly innovative, especially in the context of more behavioral neuroscience-based assays. All-in-all, I found this to be an exceptionally strong set of experiments.

Weaknesses:

No obvious weaknesses were identified by this reviewer.

# Author comment: Author response

**DOI:** 10.7554/eLife.106826.3.sa4

**Authors:** Nanci Winke, Frank Aby, Daniel Jercog, Coline Riffault, Rabia Bouali-Benazzouz, Juliette Viellard, Delphine Girard, Zoe Grivet, Marc Landry, Laia Castell, Emmanuel Valjent, Stephane Valerio, Fossat Pascal, Cyril Herry

**Affiliations:** INSERM, Neurocentre Magendie, University Bordeaux Bordeaux France; Institut des Maladies Neurodégénératives-CNRS UMR 5293 Bordeaux France; INSERM, Neurocentre Magendie, University Bordeaux Bordeaux France; INSERM, Neurocentre Magendie, University Bordeaux Bordeaux France; Institut des Maladies Neurodégénératives-CNRS UMR 5293 Bordeaux France; Institut des Maladies Neurodégénératives-CNRS UMR 5293 Bordeaux France; INSERM, Neurocentre Magendie, University Bordeaux Bordeaux France; Institut des Maladies Neurodégénératives-CNRS UMR 5293 Bordeaux France; CNRS and University of Bordeaux Bordeaux France; Department of Psychological and Brain Sciences, Krieger School of Arts and Sciences, Johns Hopkins University, Baltimore, Maryland 21218, USA Baltimore, Maryland 21218 United States; Institute for Neurosciences of Montpellier Montpellier France; INSERM, Neurocentre Magendie, University Bordeaux Bordeaux France; Institut des Maladies Neurodégénératives-CNRS UMR 5293 Bordeaux France; INSERM, Neurocentre Magendie, University Bordeaux Bordeaux France

The following is the authors’ response to the original reviews.

**Note added by the authors, October 2026:**

In preparing the data deposit for the Version of Record we carried out a figure-level traceability audit of all 101 panels of the paper, recomputing every published statistic from the value table that produced the plot. This identified a small number of corrections, which are being made in the Version of Record.

The substantive one is in Figure 6d. The CS × opsin interaction was reported as F(1,12) = 10.91, P = 0.0063; the correct value is F(1,12) = 19.87, P = 0.0008. The analysis as stored declared the repeated-measures factor on the opsin rather than on the CS, which is not the design: opsin is a between-subjects factor. The data, the group means and the effect size are unchanged, the corrected effect is stronger rather than weaker, and the conclusion — that photoactivation of the SST+ vlPAG–RVM pathway abolishes fear-induced analgesia — is unaffected.

In Figure 1—figure supplement 3, panels e and g have been redrawn so that each bar equals the ratio of the cell counts printed on it; the eight percentages concerned have been corrected in the figure and in the legend. The cell counts themselves are unchanged, and no statistical test is applied to these panels.

The remaining corrections are to reported details: the names of the statistical tests in the legends of Figures 1f and 4e, the number of cells in Figure 4d (n = 12) and in Figure 5f right n = 8, and the JAX stock number of the Sst-IRES-Cre line (013044).

The source data behind every quantitative panel of the paper are openly deposited in the Dryad Digital Repository: https://doi.org/10.5061/dryad.63xsj3vj3

**Public Reviews:**

**Reviewer #1 (Public review):**

Summary:

In the manuscript by Winke et al, the authors present evidence that fear-induced analgesia is mediated by somatostatin projection cells from the vlPAG to the RVM. This study uses a mouse model of fear-induced analgesia, and incorporates optogenetic circuit manipulation with behaviour and electrophysiology to gain a meaningful insight into a novel circuit involved in fear-induced analgesia.

Strengths:

(1) This is a well-constructed study with appropriate controls and analyses.

(2) Alternative interpretations of the data are systematically considered and eliminated via rational experiments. The authors are commended for a nice piece of experimental work.

(3) The vlPAG is a known region of pain modulation, and this study adds valuable insight to the circuit involved in fear-associated analgesia.

We are very thankful to the referee for these positive comments.

Weaknesses:

(1) Only male mice are included in this study.

We thank the reviewer for this point. We used only males in this first study for practical reasons to work with a population as homogeneous as possible. However, taking sex differences in biological mechanisms into account, we included this restriction in the summary and discussion

(2) Animals are excluded from analyses based on clearly defined criteria, but it is not clear how many mice were excluded from each group.

We thank the reviewers for raising this point. As stated in the Methods, we applied strict inclusion criteria for mice undergoing the hot-plate test, specifically a discrimination index ≥ 0.4 and a conditioning index ≥ 0.3. Using these criteria, 23% of wild-type mice were excluded for failing to meet the discrimination criterion. In the transgenic groups, an average of 20% of mice failed to meet the learning criteria, and an additional 12% were excluded due to incorrect opsin injection or misplaced optic fiber placement.

(3) The authors implement a pain sensitivity assay that involves a hot plate with progressively increasing temperature. The time to nociceptive responses is reported. Without reporting the actual temperature at which the mice respond, it makes it difficult to compare nociceptive responses to previously published work (which typically use a defined and static hotplate temperature).

We thank the reviewer for this comment. We provided this information related to the actual temperature of the nociceptive response in the original manuscript in supplementary figures 1, 2 and 5.

(4) The authors present evidence that inhibition of SST vlPAG cells enhances spinal nociceptive electrophysiological responses, but the corresponding pain sensitivity is not altered (Figure 2, CS- condition). The reason for the discrepancy between electrophysiological and behavioural responses is not clear.

We believe this comment arises from a misunderstanding of our results. In our study, inhibiting SST+ vlPAG cells did not increase nociceptive electrophysiological responses. Instead, it decreased spinal nociceptive transmission, as evidenced by reduced nociceptive field potentials and WDR responses in Figure 4c,e. Consistent with this electrophysiological effect, photoinhibition of SST+ vlPAG cells also produced behavioral analgesia, as evidenced by increased nociceptive response latency in the hotplate test under both CS− and CS+ conditions (Figure 2f). Therefore, our electrophysiological and behavioral findings are not contradictory but instead support the conclusion that inhibiting SST+ vlPAG cells reduces pain sensitivity regardless of defensive state. We will revise the text to clarify this point.

**Reviewer #2 (Public review):**

Summary:

Wenke et al. investigated the role of vlPAG somatostatin-expressing neurons in the mediation of analgesia during defensive states. A newly developed paradigm of cued fear-conditioned analgesia, which consists of a combination of an auditory fear retrieval session and a pain test, was used to evaluate this cell population's contribution to fear-mediated analgesia. Optogenetic manipulation of vlPAG SST+ neurons modulated the responses to a nociceptive cue (Hot Plate) presented concomitantly with an aversively conditioned tone. At the same time, alterations in the freezing levels could be observed during optogenetic activation of vlPAG SST+ neurons. In order to disentangle the impact of these cells on analgesia from their impact on the expression of defensive behaviors, the authors performed electrophysiological recordings from the dorsal horn in the spinal cord of anesthetized mice. A vlPAG-RVM-DH pathway was identified to trigger nociceptive C-fibers upon optic activation of the RVM. Finally, pathway-specific activation of SST+ vlPAG-RVM neurons could abolish CS-induced analgesia.

Strengths:

The study addresses a relevant topic, that is, brainstem circuits for pain-modulatory mechanisms as part of defensive states evoked by threat. This is important because the circuit mechanisms underlying pain are still not fully understood, and defining molecular markers of cellular circuit substrates may support the identification of potential pharmaceutical targets in treating pain. The authors confirm a previous study in that a somatostatin-positive cellular population presents a crucial vlPAG circuit element mediating anti-nociceptive effects. Key novelty aspects of the present study are the demonstration that these neurons seem to play a role specifically in threat-induced analgesia. This was possible by the elegant design and application of a novel fear analgesia paradigm, combined with cell- and pathway specific optogenetics.

We thank the referee for such positive feedback.

Weaknesses:

Despite the convincing and rigorous experimental approach, the study leaves some interpretational room when it comes to the proposed circuit mechanism. This could either be addressed by additional experiments or by more discussion of alternative circuit layouts.

Major Comments:

(1) The paper by Zhang et al. (https://pubmed.ncbi.nlm.nih.gov/36641028/), which identified a role for vlPAG SOM+ neurons in mediating anti-nociception in neuropathic pain, needs to be referenced and its results discussed, if not reconciled. While functionally, both studies find an analgetic role of vlPAG SOM+ neurons projecting to the RVM, Zhang et al., using slice physiology, characterize those neurons as glutamatergic. In Figure 4E of Zhang et al. they find general (fear-independent) analgetic effects with PAG-RVM specificity by performing chemogenetic experiments.

We thank the reviewer for highlighting this important point. We agree that the study by Zhang et al. is highly relevant and should be discussed in the revised manuscript. Their work shows that inhibiting vlPAG SST/SOM neurons with chemogenetic methods produces analgesia in a neuropathic pain model, and in our study, we similarly found that inhibiting SST+ vlPAG neurons increases hotplate response latency (Figure 2f), which aligns with an analgesic effect. Additionally, we observed that activating SST+ vlPAG neurons suppresses fear-conditioned analgesia.

At the same time, there are important differences between the two studies that may explain the differences in interpretation. First, the behavioral paradigms are not identical. Zhang et al. used a hotplate protocol where animals were directly exposed to a nociceptive temperature, whereas in our study, we used a progressive temperature ramp and explicitly compared responses during a conditioned stimulus (CS+) and a non-conditioned control stimulus (CS−). These controls were important for us to distinguish fear-specific effects from more general effects related to stress, arousal, sensitization, or other non-associative processes.

Second, the two studies differ in experimental context. Zhang et al. examined this circuit in a neuropathic pain model, whereas our study focused on acute nociceptive processing and fear-conditioned modulation of pain. We therefore believe that the apparent discrepancy might reflect differences in pain state and behavioral context, rather than a direct contradiction.

Finally, Zhang et al. showed in slice recordings that SST+ vlPAG neurons provide excitatory input to RVM neurons. This is an important finding that we now address in the revised manuscript. At the same time, because the RVM contains heterogeneous neuronal populations with different projection targets and functions, these recordings alone do not prove that all recorded RVM neurons are part of the descending pathway controlling spinal nociception. Therefore, we have revised the Discussion to explicitly acknowledge Zhang et al. and to emphasize both the similarities and differences between the two studies.

It can be argued that in addition to the two functionally distinct inhibitory SOM subtypes hypothesized by Winke et al., there is another, excitatory subpopulation. Also, the different experimental conditions chronic vs. acute pain, non-threat vs. fearful cues/contexts may recruit different vlPAG SOM+ populations. All of this is conceivable, yet I wonder whether the contrasting findings could more parsimoniously be reconciled. The author's own results presented here in Supplementary Figure 3 suggests that SOM+ vlPAG cells are colocalizing with glutamate and thus could also be excitatory. In addition to this rather complementary piece of evidence, a more extensive characterization of vlPAG neurons using IHC and slice physiology would be needed to justify the unambiguous identification of their inhibitory nature.

We thank the reviewer for this thoughtful comment. We agree that our current data do not support a definitive conclusion that all SST+ vlPAG neurons are inhibitory. As the reviewer notes, our Supplementary Figure 3 shows that SST+ vlPAG cells can also co-localize with glutamatergic markers, which is consistent with the possibility of cellular heterogeneity within this population. We also agree that different experimental conditions, such as chronic versus acute pain and non-threatening versus fear-related contexts, may activate different SST+ vlPAG subpopulations.

Our intention was not to claim that SST+ vlPAG neurons constitute a uniform inhibitory population, but rather that SST+ cells are strongly represented among inhibitory neurons in the vlPAG. We agree, however, that more detailed characterization, including additional immunohistochemical analyses and slice physiology, is necessary to more definitively determine the neurotransmitter phenotype and functional connectivity of these neurons. We have therefore revised the text to temper our interpretation and to explicitly acknowledge the likely heterogeneity of SST+ vlPAG neurons, including the possibility of an excitatory subpopulation. We therefore modified the discussion accordingly:

“Our results align with the parallel inhibition- excitation model, where inhibitory and excitatory cells form two distinct, parallel descending pathways for pain modulation.

Indeed, previous research demonstrated the presence of an inhibitory pathway projecting throughout the PAG–RVM-spinal cord dorsal horn neuraxis. Our results complement this study by suggesting that one of these previously proposed parallel pathways is mediated by SST+ vlPAG cells and has a functional role in mediating analgesia. At the same time, our data indicate that vlPAG SST neurons are heterogeneous, with approximately one-third of these cells co-localizing with excitatory markers. Together with the recent observation that excitatory SST+ vlPAG neurons project to the RVM (Zhang et al., 2023), this raises the possibility that a subset of long-range SST+ vlPAG neurons contributes to an excitatory descending pathway within the PAG–RVM–spinal dorsal horn neuraxis. By contrast, local GABAergic SST+ vlPAG neurons may participate in local circuit mechanisms related to defensive-state expression, including freezing. Further anatomical and functional studies will be required to resolve these possibilities.”

In the absence of a direct identification of these cells exclusively releasing GABA, an alternative explanation should be considered. What about looking at vlPAG SOM+ neurons as a putatively mixed bag of local, inhibitory interneurons and long-range, RVM-projecting excitatory cells? This model would then open up interesting questions as to the actual function of somatostatin as a modulator of vlPAG circuit activity and associated function, and from my perspective, would nicely fit into the view of PAG circuits as integrators of complex survival responses.

We thank the reviewer for this insightful suggestion and agree that, in the absence of direct evidence that vlPAG SOM+/SST+ neurons are exclusively GABAergic, an alternative interpretation should be considered. In particular, we agree that this population may be heterogeneous and could include both local inhibitory interneurons and long-range excitatory neurons projecting to the RVM. We believe this is an important and constructive framework for interpreting our data, and we have revised the Discussion accordingly. In the revised text, we now explicitly acknowledge the likely heterogeneity of vlPAG SST+ neurons and discuss the possibility that distinct local and long-range SST+ subpopulations may contribute differently to defensive-state regulation and descending pain modulation. We agree with the reviewer on this point and have modified the discussion accordingly (see point above).

(2) "Our data indicate that the optogenetic inhibition of SST+ vlPAG cells promotes analgesia irrespective of the animal's defensive state. In contrast, the optogenetic activation of long-range SST+ vlPAG cells that project to the rostral ventromedial medulla (RVM) abolishes the analgesia mediated by fear behavior." (lines 32-35). Consider toning down these conclusions, as contrasting activation with inhibition of two different (though overlapping) populations cannot be fully conclusive. Alternatively, a pathway-specific (vlPAG-RVM) inhibitory experiment could help to fully understand the circuit mechanism and verify the necessity of these neurons.

We thank the reviewer for raising this point. We agree that inhibition of the entire SST+ vlPAG population and activation of the long-range SST+ vlPAG neurons projecting to the RVM population are not directly equivalent manipulations. Our conclusion was intended at the level of observed functional effects: inhibition of SST+ vlPAG neurons promotes analgesia regardless of the defensive state, while activating long-range SST+ vlPAG neurons projecting to the RVM suppresses fear-conditioned analgesia. This occurs regardless of whether the SST vlPAG neurons are excitatory or inhibitory. To address the excitatory or inhibitory nature of SST vlPAG neurons, we have revised the discussion to include a reference to the Zhang et al study.

(3) Despite an overall very thorough reporting style, some information is missing from the manuscript:

(a) In Figures 2d and f, what are the freezing levels during optogenetic manipulation? From Figure 3d, one can expect that freezing is inhibited during the hot plate test, which could bias the NC response towards shorter latencies.

We thank the reviewer for this important comment. As shown in Figure 1e, we previously quantified freezing both at CS onset and at the time of the nociceptive response in the hot plate test. These analyses indicate that freezing levels at the time of the nociceptive response do not differ between the CS+ and CS− conditions. Therefore, the variation in hot plate response latency is unlikely to be due to differences in freezing at the time of response.

We acknowledge, however, that freezing was not directly measured during optogenetic manipulation in this experiment. Based on the temporal profile of freezing shown in Figure 1e, we still consider it unlikely that the effect of optogenetic manipulation on nociceptive latency is mainly caused by a change in freezing behavior.

(b) In Figure 5, the histological experiment showing the vlPAG-to-RVM pathway is presented by a qualitative image only. Here, some quantification would strengthen the finding.

We thank the reviewer for this comment. The aim of the histological experiment in Figure 5 was to provide qualitative anatomical evidence that vlPAG projections reach the RVM and are positioned in close apposition to spinally projecting RVM neurons. We did not intend this experiment to serve as a quantitative characterization of connectivity. We agree that a more systematic quantification would be informative, but this would require additional dedicated experiments beyond the scope of the present manuscript.

(c) In Figures 6 c and d "Consistently, activation of the SST+ vlPAG-RVM pathway during CFCA had no impact on CS-presentation, whereas the same manipulation performed during CS+ blocked the increase in NC response latency compared to GFP controls." (line 194-196). Is it possible that the NC response cannot be any lower than the one during CS-, thus constituting a floor effect?

We are thankful to the reviewer for this important point. We agree with the reviewer that this is indeed a possibility. We have added a sentence in the discussion to acknowledge this limitation.“Another possibility is that our nociceptive test with a slow ramp of temperature induces a floor effect on nociceptive response latency, which may limit the detection of further decreases in latency under certain conditions.”

(c) Connected to major point 1- this experiment is important for defining the circuit mode and therefore should be as convincing as possible. However, for the colocalization experiment in Supplementary Figure 3, the methodological description is missing and thus makes it hard to comprehend how this data set was generated (how many data points, etc.). The visual depiction of the results is non-standard and not easily graspable. Consider e.g., a Venn diagram.

We apologize for this omission in the original manuscript. We have now provided this methodological information in the method section. We have now expanded the description of these data in the figure legend to ease the comprehension of the figure.

**Reviewer #3 (Public review):**

Summary:

Conditioned analgesia refers to the ability of a learned fear cue to suppress pain-related behavior and neural activity. Understudied, the authors developed a novel conditioned analgesia procedure in which a cue that had been paired or unpaired with shock was played while a hot plate increased temperature. Compared to several control conditions, the authors found increased latency to a nociceptive response (paw licking). The authors identified somatostatin neurons in the periaqueductal gray as a likely mediator of the behavior. They then showed that: (1) stimulating vlPAG-SST neurons blocked nociceptive response latency increases to the CS+, (2) stimulating vlPAG-SST neurons suppressed fear retrieval freezing, (3) stimulating vs. inhibiting vlPAG-SST neurons drove opposing modulation of c-fibers and Aδfibers, (4) direct-projecting vlPAG SST neurons modulate freezing while RVM-projecting vlPAG SST neurons modulate conditioned analgesia.

Strengths:

These experiments have many strengths. The behavioral assay is chief among them. The assay is robust and controls for confounding factors to reveal a repeatable effect of a shock-paired cue to delay nociceptive responding. The optogenetic experiments provide the correct level of temporal precision, given the authors' time-specific interest in cued responding. Combining neuronal manipulations with spinal recordings is particularly innovative, especially in the context of more behavioral neuroscience-based assays. All-in-all, I found this to be an exceptionally strong set of experiments.

Weaknesses:

No obvious weaknesses were identified by this Reviewer.

**Recommendations for the authors:**

**Comments from Reviewing Editor:**

Summary

Three reviewers have assessed your manuscript on vlPAG somatostatin pathways contributing to conditioned analgesia. Conditioned analgesia refers to the ability of a learned fear cue to suppress pain-related behavior and neural activity. Understudied, the authors developed a novel conditioned analgesia procedure in which a cue that had been paired or unpaired with shock was played while a hot plate increased temperature. Compared to several control conditions, the authors found increased latency to a nociceptive response (paw licking). The authors identified somatostatin neurons in the periaqueductal gray as a likely mediator of the behavior. They then showed that: (1) stimulating vlPAG-SST neurons blocked nociceptive response latency increases to the CS+, (2) stimulating vlPAG-SST neurons suppressed fear retrieval freezing, (3) stimulating vs. inhibiting vlPAG-SST neurons drove opposing modulation of c-fibers and Aδ-fibers, (4) direct-projecting vlPAG SST neurons modulate freezing while RVM-projecting vlPAG SST neurons modulate conditioned analgesia.

Strengths

All three reviewers converged on multiple strengths. The assay developed was seen to be novel, rigorous, and included a variety of controls that convincingly demonstrated conditioned analgesia. Focusing on the ventrolateral periaqueductal gray, and more specifically on somatostatin-expressing cells, made prior sense, and the results more than justified this selection. Approaching the vlPAG and circuits with many converging methods provided further, compelling evidence for a role in conditioned analgesia.

Weaknesses

Specific weaknesses are described in the individual reviews. Generally, the following weaknesses were identified. The study only used male mice, a choice that should be better justified. Animals were reasonably excluded from analysis, but the final group ns for analyses were not always clear. Some statistical results lacked clarity. The relevance of these findings to prior work (particularly Zhang et al. 2023, Journal of Pain) was not always described. Relatedly, the results would be better contextualized by appreciating and describing the likely diversity of somatostatin functional types and projection types.

Recommendations

(1) Provide rationale for only using male mice, discuss the limitation of the exclusion of females, and note that male mice were the subjects in the abstract.

Thank you for this recommendation, we have mentioned this information in the abstract and in the discussion. We have also mentioned the limitations of not including female mice in the abstract and the discussion of the revised manuscript.

(2) Complete final report ns for each statistical analysis. If you have not already done so, please include full statistical reporting including exact p-values wherever possible alongside the summary statistics (test statistic and df) and, where appropriate, 95% confidence intervals. These should be reported for all key questions and not only when the p-value is less than 0.05 in the main manuscript.

An extended table with all statistical tests and analysis for all figures has been provided in sup Table 1.

(3) Include example videos of CFCA sessions, demonstrating optogenetic effects.

We understand the editor’s request to include video material illustrating the behavioral responses. However, we would prefer not to include such videos in the manuscript, in accordance with our institution's guidelines and recommendations on the dissemination of animal experimentation footage. Importantly, all behavioral sessions were systematically video-recorded from both sides of the apparatus, allowing detailed offline analysis of the animals’ responses. These recordings were carefully examined by an experienced experimenter to assess nociceptive behaviors, including jumping responses and licking of the stimulated hindpaw. This procedure ensured a reliable and accurate evaluation of pain-related behavioral reactivity. While the videos themselves cannot be included in the manuscript for the reasons mentioned above, we believe that the behavioral scoring procedures described in the Methods section provide a clear and rigorous description of how these responses were assessed. In addition, Figure 1 includes an example image illustrating hindpaw licking behaviour, which is typically more subtle and more difficult to identify than jumping responses. We therefore believe that this visual example, together with the detailed description of the scoring procedure and the quantitative data provided, adequately supports the interpretation of the behavioural results.

(4) Provide summary expression and ferrule placement figures.

We thank the editor for this comment. We have now included schematic summaries of fiber placements for both SST and VIP mice used in this study, based on histological verification (Supplementary Figures 10 and 11). Representative images of viral expression are also provided (Figure 2a, Supplementary Figure 7b and f).

(5) Detail how behavior judgments were made.

We thank the editor for emphasizing this important methodological point. During all behavioral sessions, mice were video-recorded simultaneously from both sides of the apparatus, allowing a comprehensive and unobstructed view of the animals’ posture and movements throughout the experiment. These recordings were subsequently analyzed offline by an experienced experimenter trained to evaluate nociceptive behaviors. Pain-related behavioral responses were assessed based on well-established indicators of nociceptive reactivity. In particular, we quantified overt escape-like reactions such as jumping, which reflects a strong aversive response to the stimulus. In addition, we evaluated more localized nociceptive behaviors directed toward the stimulated limb, including licking of the hindpaw. These measures are commonly used in rodent pain assays and provide reliable behavioral readouts of nociceptive sensitivity. The combination of bilateral video recordings and expert behavioral scoring ensured that both subtle and robust nociceptive responses could be accurately detected and categorized during the analysis.

(6) Provide the temperature at which nociceptive responses were initiated. Check grammar and references.

The temperature at which nociceptive responses were initiated were originally reported in Supplementary Figure 1, 2 and 5.

**Reviewer #1 (Recommendations for the authors):**

(1) The authors use optogenetic manipulation of SST activity in the vlPAG to show that this cell type is involved in fear-induced analgesia. They include a valuable control to show that manipulation of another inhibitory cell type (VIP) also does not impact analgesia. It would be helpful to know the expression level of VIP cells in the vlPAG. Is this a predominant inhibitory projection cell in the vlPAG (besides SST)?

We thank the reviewer for pointing this. While we did not quantify the expression level of VIP+ cells in the vlPAG in the present study, available data suggest that this population is relatively sparse compared to other inhibitory cell types. In particular, reference to the Allen brain atlas indicates that VIP gene expression in the vlPAG is limited and primarily localized around the fourth ventricle, within the lateral and ventrolateral PAG, rather than broadly distributed across the region. Consistent with this, we provide an example of viral expression in VIP-Cre mice in Supplementary Figure 7f, illustrating the restricted distribution of VIP+ neurons in the vlPAG. We have also provided a summary of ferrules placement for SST and VIP mice used in our study in Supplementary Figures 11 and 10, respectively.

(2) The numbers of animals dropped from each experiment should be indicated - perhaps on the statistics table?

We thank the reviewer for pointing this.

As stated in the Methods, we applied strict inclusion criteria for mice undergoing the hot-plate test, specifically a discrimination index ≥ 0.4 and a conditioning index ≥ 0.3. Using these criteria, 23% of wild-type mice were excluded for failing to meet the discrimination criterion. In the transgenic groups, an average of 20% of mice failed to meet the learning criteria, and an additional 12% were excluded due to incorrect opsin injection or misplaced optic fiber placement.

(3) Line 105: "...,which activity..." change to "..., whose activity..."

Done

**Reviewer #2 (Recommendations for the authors):**

(1) Please also provide absolute temperature values of the nociceptive response threshold.

The temperature at which nociceptive responses were initiated was originally reported in Supplementary Figure 1, 2 and 5.

(2) It would be nice to see an example video of a CFCA session (with and without optogenetic manipulation).

We understand the editor’s and reviewer’s request to include video material illustrating the behavioral responses. However, we would prefer not to include such videos in the manuscript, in accordance with our institution's guidelines and recommendations on the dissemination of animal experimentation footage. Importantly, all behavioral sessions were systematically video-recorded from both sides of the apparatus, allowing detailed offline analysis of the animals’ responses. These recordings were carefully examined by an experienced experimenter to assess nociceptive behaviors, including jumping responses and licking of the stimulated hindpaw. This procedure ensured a reliable and accurate evaluation of pain-related behavioral reactivity. While the videos themselves cannot be included in the manuscript for the reasons mentioned above, we believe that the behavioral scoring procedures described in the Methods section provide a clear and rigorous description of how these responses were assessed. In addition, Figure 1 includes an example image illustrating hindpaw licking behaviour, which is typically more subtle and more difficult to identify than jumping responses. We therefore believe that this visual example, together with the detailed description of the scoring procedure and the quantitative data provided, adequately supports the interpretation of the behavioural results.

(3) Please provide a schematic summary of fiber placements and opsin expressions confirmed by histological examinations.

We thank the reviewer for this comment. We have now included schematic summaries of fiber placements for both SST and VIP mice used in this study, based on histological verification (Supplementary Figures 10 and 11). Representative images of viral expression are also provided (Figure 2a, Supplementary Figure 7b and f).

(4) "Valid nociception readout responses included jumping or licking the hindpaw." (Line 453). How was this evaluated- manually or automated, blinded etc.?

We thank the reviewer for emphasizing this important methodological point. During all behavioral sessions, mice were video-recorded simultaneously from both sides of the apparatus, allowing a comprehensive and unobstructed view of the animals’ posture and movements throughout the experiment. These recordings were subsequently analyzed offline by an experienced experimenter trained to evaluate nociceptive behaviors. Pain-related behavioral responses were assessed based on well-established indicators of nociceptive reactivity. In particular, we quantified overt escape-like reactions such as jumping, which reflects a strong aversive response to the stimulus. In addition, we evaluated more localized nocifensive behaviors directed toward the stimulated limb, including licking of the hindpaw. These measures are commonly used in rodent pain assays and provide reliable behavioral readouts of nociceptive sensitivity.The combination of bilateral video recordings and expert behavioral scoring ensured that both subtle and robust nociceptive responses could be accurately detected and categorized during the analysis.

(5) Line 226 REF33 doesn't seem to fit.

The reference list has been updated. Related to this section in which we discuss the disinhibition mechanisms inducing nociception in chronic stress mice. We have cited the work of Samineni et al., 2015 (reference 15) and Tovote el al., (reference 23) both related to these disinhibition mechanisms.

Full sentence for reference 33 (now 35): “Two independent previous studies found that long-range inhibitory inputs from the central medial amygdala contact inhibitory cells within the vlPAG, implicated in different roles: the modulation of fear behavior (23) and nociceptive transmission (35)”.

Ref 35 - Yin, W. et al. A Central Amygdala–Ventrolateral Periaqueductal Gray Matter Pathway for Pain in a Mouse Model of Depression-like Behavior. Anesthesiology 132,1175–119 (2020)

(6) Some minor language, semantic, and grammatical flaws.

The manuscript has been evaluated for language, semantic and grammatical flaws
